# Sensitivity Analysis of the NPM-ALK Signalling Network Reveals Important Pathways for Anaplastic Large Cell Lymphoma Combination Therapy

**DOI:** 10.1371/journal.pone.0163011

**Published:** 2016-09-26

**Authors:** Antoine Buetti-Dinh, Thomas O’Hare, Ran Friedman

**Affiliations:** 1 Department of Chemistry and Biomedical Sciences, Linnæus University, Kalmar, Sweden; 2 Linnæus University Centre for Biomaterials Chemistry, Linnæus University, Kalmar, Sweden; 3 Institute of Computational Science, Faculty of Informatics, Università della Svizzera Italiana, Lugano, Switzerland; 4 Swiss Institute of Bioinformatics, Lausanne, Switzerland; 5 Huntsman Cancer Institute, The University of Utah, Salt Lake City, United States of America; 6 Division of Hematology and Hematologic Malignancies, The University of Utah, Salt Lake City, United States of America; Queen’s University Belfast, UNITED KINGDOM

## Abstract

A large subset of anaplastic large cell lymphoma (ALCL) patients harbour a somatic aberration in which anaplastic lymphoma kinase (ALK) is fused to nucleophosmin (NPM) resulting in a constitutively active signalling fusion protein, NPM-ALK. We computationally simulated the signalling network which mediates pathological cell survival and proliferation through NPM-ALK to identify therapeutically targetable nodes through which it may be possible to regain control of the tumourigenic process. The simulations reveal the predominant role of the VAV1-CDC42 (cell division control protein 42) pathway in NPM-ALK-driven cellular proliferation and of the Ras / mitogen-activated ERK kinase (MEK) / extracellular signal-regulated kinase (ERK) cascade in controlling cell survival. Our results also highlight the importance of a group of interleukins together with the Janus kinase 3 (JAK3) / signal transducer and activator of transcription 3 (STAT3) signalling in the development of NPM-ALK derived ALCL. Depending on the activity of JAK3 and STAT3, the system may also be sensitive to activation of protein tyrosine phosphatase-1 (SHP1), which has an inhibitory effect on cell survival and proliferation. The identification of signalling pathways active in tumourigenic processes is of fundamental importance for effective therapies. The prediction of alternative pathways that circumvent classical therapeutic targets opens the way to preventive approaches for countering the emergence of cancer resistance.

## Introduction

Receptor tyrosine kinases (RTKs) located on the cell surface regulate diverse cellular processes involved in cell survival and proliferation. Mutations that alter RTK function can lead to the development of a variety of cancer types. Accordingly, several RTKs are targets for oncological drugs, and others are studied as potential targets. The anaplastic lymphoma kinase (ALK) is an RTK involved in the development of the nervous system. Midkine, pleiotrophin [1], and more recently heparin [2] were shown to be ALK ligands. Chromosomal rearrangements produce oncogenic fusions of ALK [3] with proteins such as the echinoderm microtubule associated protein-like 4 (EML4) [4] and nucleophosmin (NPM) [5]. The aberrant activity of ALK fusions, as a consequence of chromosomal rearrangements, leads to the development of multiple malignancies such as non-small cell lung cancer (NSCLC) [6] and ALK-positive ALCL [7] (the latter representing ∼ 50–80% of all ALCLs [8] of which in ∼ 85% ALK is fused with NPM [9]). Two ALK inhibitors, crizotinib (Xalkori^®^) [10] and ceritinib (Zykadia^®^) [11] are approved as treatment against NSCLC driven by rearranged ALK. Crizotinib is also used to treat patients with anaplastic large cell lymphoma (ALCL) that is refractory to chemotherapy [12]. Numerous other ALK inhibitors are at various stages of clinical or preclinical development [13, 14].

One of the difficulties in treating cancers is the development of drug resistance. In many cases of targeted treatment, tumours develop resistance mechanisms that enable them to overcome the effects of the drug. Amino acid mutations in the ALK kinase domain can confer resistance by weakening the binding of a therapeutic inhibitor [6, 15–25]. This enables ALK to maintain kinase activity that sustains cancer development despite high inhibitor concentrations. Resistance-conferring mutations appear to be the prevalent mechanism of treatment failure in ALCL relapsed patients, though this conclusion is based on very few cases [12].

Another important cause of drug resistance is the recruitment of alternative signalling routes. These allow tumour cells to bypass signalling pathways blocked by therapeutic inhibitors. Thereby, the tumour maintains the activity of cellular functions necessary for growth without relying on the inhibited drug target. The key factors underlying this phenomenon are the redundancy and pleiotropy of biological signalling pathways, and the flexible character of biological signalling that allows dynamic adaptation to changing conditions [26, 27]. Intricate signalling networks have been shown to mediate resistance to ALK inhibitors in ALK-positive NSCLC [20, 28].

Several efforts have been made to understand the biological pathways underlying ALK signalling and counter ALK fusion’s deregulated activity in NSCLC and ALCL. In ALK-positive ALCL, cellular components involved in the downstream signalling of ALK include the Janus kinase 3 (JAK3) / signal transducer and activator of transcription 3 (STAT3) and interacting partners; and phospholipase C (PLC-*γ*) and secondary messengers together with class-Ia phosphoinositide 3-kinase (PI3K) / protein kinase B (AKT). These pathways are known to promote tumour growth by influencing proliferation, cell survival, growth, and motility [29–31]. Another crucial cascade involved in ALK signalling is the Ras / mitogen-activated ERK kinase (MEK) / extracellular signal-regulated kinase (ERK) pathway and its associated effectors that enhance tumour growth in many cancer models. Importantly, aberrant ALK activity induces activation of cellular pathways that are strongly interconnected and overlapping, meaning that the ALK interaction network is strongly pleiotropic and redundant [32]. Tumour development is consequently expected to be driven by a complex, nonlinear regulation, making it challenging to predict which signalling components in the NPM-ALK network need to be inhibited by targeted inhibitors in order to impair tumour growth.

To improve the current therapies against ALK-positive tumours, it may not be sufficient to develop ALK inhibitors that counter resistance mutations. Rather, it is also important to identify critical signalling components in the ALK interaction network that control pathways involved in cancer development and particularly resistance. It has recently been demonstrated in patient-derived cell models that such an approach has potential in various forms of NSCLC [33]. These components represent crucial control points of the ALK signalling pathway and their combined targeting is likely to be of fundamental importance for efficient therapies that circumvent tumour resistance.

Here we apply numerical simulation methods to perform sensitivity analysis of the NPM-ALK network. We show that with the available information on the NPM-ALK network topology, it is possible to elucidate the signalling routes that are crucial for cancer-driving processes. This enables us to pinpoint alternative signalling paths used as escape routes by drug-resistant tumours, and suggest targets for combination therapies.

## Results and Discussion

### Coarse-Grained Simulations: Identification of Active Signalling Pathways in the NPM-ALK Network

**Fig 1:**
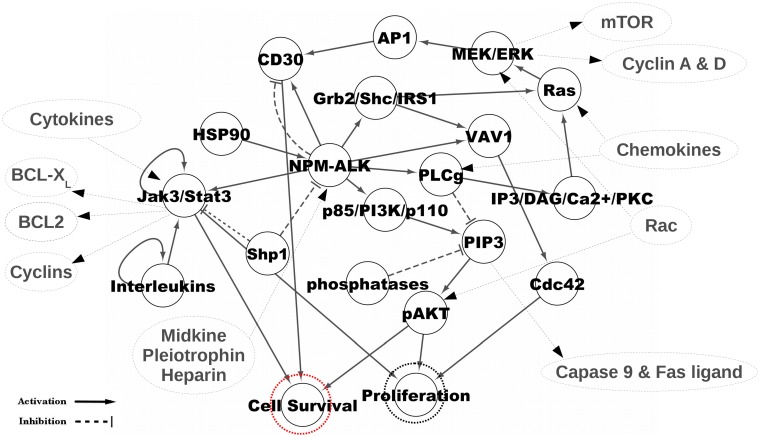
NPM-ALK network scheme. Nodes and links in black represent the core network whose components have been considered in our simulations. The surrounding components (nodes and arrows represented with grey dotted lines) indicate factors that influence the core network in different contexts and are not considered in the simulations. In the core network, the relevance of different nodes for signalling in disease development are linked by activating and inhibiting network’s links, indicated with → and ⊣, respectively. “Interleukins” includes IL-2, IL-4, IL-7, IL-9, IL-15, IL-21 and IL-22 [34–36], while “Phosphatases” refers to lipid phosphatases such as the phosphatase and tensin homologue, which converts phosphatidylinositol-3,4,5-triphosphate (PIP3) back to phosphatidylinositol-4,5-bisphosphate (PIP2) [37].

The NPM-ALK network (Fig 1) was first investigated by simulating all combinations of network states where each node could adopt one of two initial activity states: *high* (*β* = 0.1) or *low* (*β* = 0.001) (see the “Methods” section for details). This model corresponds to tumours or cell lines where the activity of certain components in the signalling network becomes higher or lower due to external perturbations such as environmental factors or internal perturbations due to gene mutations or epigenetic alterations.

### Identification of the Most Important Contributors to Cell Survival and Proliferation

**Fig 2:**
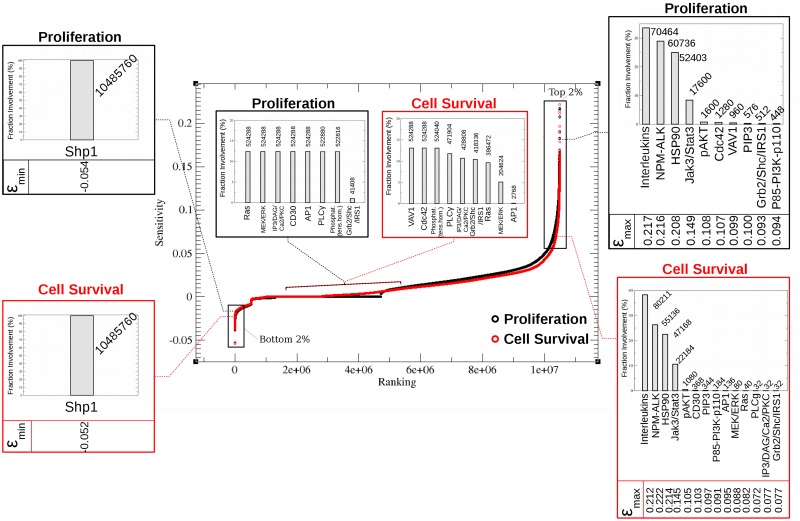
Sensitivity profile of the network. Sensitivity of the endpoints (“Cell Survival” and “Proliferation”, encircled in the network representation Fig 1 in red and black, respectively) is calculated according to [38, 39] and represented in red and black data points in the central plot. Each point in the central plot corresponds to one of $2^{20}\cdot \frac{20}{2}\;\approx \;10^{7}$ states that differ from each other in exactly one node (recall that each node may assume a value of *low* or *high* activity). This enables the calculation of the sensitivity of every endpoint with respect to each node by comparing all pairs of states systematically. Following this step, the states that contributed the most to the sensitivity (the top- or bottom-2% regions) were examined to view how often each node is associated with the outcome. The results of this calculation are presented in the bar plots. *ε*_*max*_ (*ε*_*min*_) indicates the maximum (minimum) sensitivity value corresponding to each bar in the plots on the right and left-hand sides. The bar plots in the middle represent the fractional involvement of the network nodes associated with negligible sensitivity (between ±0.005).

The first aim in the analysis of the simulations was to identify the states (combinations of nodes with different activity levels, denoted as the network’s “*control nodes*”) that most strongly influence the pathological outcome, *i.e.*, cell proliferation and survival (denoted as the network’s “*endpoints*”). Sensitivity analysis was therefore performed, and the states were sorted according to their influence on proliferation and cell survival (central plot in Fig 2). The nodes with high or low (*i.e.*, negative) sensitivity values with respect to the endpoints constitute control points of the network for tumour development.

To identify the nodes that affect proliferation and cell survival most significantly, the states that correspond to the highest (top-2%) or lowest (bottom-2%) values of the sensitivity profile were identified. The more often a node is associated with high or low (*i.e.*, negative) sensitivity, the more influential it is. Conversely, the nodes linked to the values present in the middle plateau of the global sensitivity profile are the nodes that least influence the network endpoints. Irrespective of the state of the other network nodes, a variation in their activity does not affect the endpoints.

The sensitivity ranking analysis reveals three principal categories of nodes. First, there are nodes that contribute to decreased endpoint activity when their own activity is increased (bottom-2% of the sensitivity ranking). These nodes have tumour suppressing capability. Only one such node was identified here, protein tyrosine phosphatase-1 (SHP1, also known as PTPN6). Upon increased activity of SHP1, both proliferation and cell survival are predicted to decrease. This outcome could be expected, since SHP1 inhibits network components close to the endpoints. The role of SHP1 has been associated with the maintenance of physiological signalling and its loss is reported to lead to uncontrolled cell growth [32] and development of anaplastic lymphoma [37, 40–43]. Its role as a tumour suppressor is thus validated.

The second category of nodes are those that are causally linked (often through indirect pathways comprising several links) to an increase of the proliferation or cell survival activities. The significance of an individual node in this subset is reflected by the number of states within the top-2% subset where the node was a determinant for endpoints control. For example, of the 209 715 states in the top-2% region, interleukins’ activity is playing a determining role in 70 464 of the cases (≈ 34%) for proliferation and 80 211 (≈ 38%) for cell survival. High activity of the interleukins IL-2, IL-4, IL-7, IL-9, IL-15, IL-21 and IL-22 is thus associated with tumourigenesis, which is in line with the current understanding of tumour biology [35, 44]. The apparent influence of interleukins on cell growth and proliferation is explained by the positive feedback on interleukins due to autocrine stimulation [35, 45, 46]. Their direct interaction with JAK3/STAT3, which is also under positive feedback [47, 48], further explains the strong influence onto the network endpoints. Thus, the network reveals that interleukins and JAK3/STAT3 can be markers of cell survival and proliferation. Interestingly, therapies based on antibodies as well as small organic compounds that target JAK/STAT reduced proliferation and cell survival in experimental cancer therapies [49–51] and played a role in the control of immune responses [52–55]. In addition, interleukins have been the focus of therapies against several types of cancer. Neutralizing antibodies were used against IL-9 resulting in a decrease of cell survival and proliferation in NPM-ALK-positive ALCL pointing out the cross-talk between interleukins and STAT/JAK signalling in ALCL [35]. More biological data will be needed to discern the correlation between the expression of individual interleukins and proliferation or cell survival. Note that it is possible to refine our model once additional experimental evidence (*e.g.*, from omics experiments) will be made available. Moreover, it will be possible to test different therapeutic strategies (*e.g.*, the selective inhibition of single interleukins or the most efficient combinations thereof) in our simulation system.

The majority of the nodes belongs to a third group—nodes that have a very low influence on the endpoints. Consequently, therapeutic treatments targeted at such nodes are not expected to benefit the patients. Nodes with negligible effects on the network endpoints (sensitivity between ±0.005) are shown in the bar plots in the middle of Fig 2. These nodes include oncogenes such as Ras and progression promoters such as PLC-*γ*. The simulations suggest a different involvement of some nodes for controlling the outcome of cell survival *versus* proliferation. A role of the Ras/MEK/ERK cascade is predicted in controlling cell survival, and of the VAV1-CDC42 (cell division control protein 42) path in controlling proliferation (further discussed in section “Network States that Promote Increased Proliferation and Cell Survival”).

The side bar plots in Fig 2 represent the importance of the network nodes based on their frequency among the top- or bottom-2% fraction of the ranked sensitivity profiles. The maximal value of the effect (sensitivity) is shown below each bar. SHP1 has minimal (most negative) sensitivity values when considering either proliferation or cell survival. Although these values are rather small in absolute terms (-0.054 and -0.052 for proliferation and cell survival, respectively), they still represent a ∼10-fold difference with respect to the nodes in the middle portions of the central plot in Fig 2. Considering the node associated with the maximal value for sensitivity, the situation is not exactly the same for proliferation (interleukins, *ε*_*max*_ = 0.217, with NPM-ALK having a very similar value *ε*_*max*_ = 0.216) and cell survival (*ε*_*max*_ = 0.222, NPM-ALK). This explains the crucial dependence of the network on NPM-ALK. Note also that interleukins were grouped together here due to insufficient information on the individual roles of each of them. If it were possible to separate the interleukin group, it is likely that NPM-ALK would be the most influential node in the network.

### Network States that Promote Increased Proliferation and Cell Survival

Computational network analysis predicts that both proliferation and cell survival are driven by the NPM-ALK → JAK3/STAT3 and the NPM-ALK → p85/PI3K/p110 → PIP3 → pAKT pathways. Only cell survival however appears to be also controlled by the pathway involving growth factor receptor–bound protein 2 (GRB) / Src homology and collagen (SHC) / insulin receptor substrate-1 (IRS1) and/or PLC-*γ* → inositol-1,4,5-triphosphate (IP3) / membrane-bound diacylglycerol (DAG) /Ca^2+^/PKC which converge to the Ras/MEK/ERK cascade. In contrast, our network analysis suggests that proliferation is maintained by the NPM-ALK → GRB/SHC/IRS1 → VAV1 → CDC42 pathway and is independent of the Ras/MEK/ERK cascade. This outcome is supported by recent experimental evidence where dual interruption of STAT3 and ERK pathways resulted in decreased proliferation and cell survival [56].

**Fig 3:**
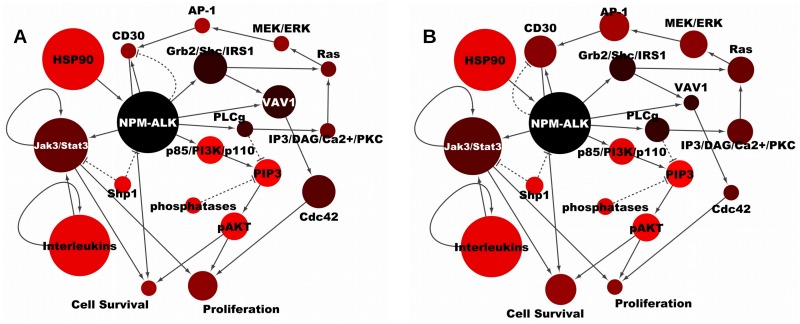
Signal flow in the NPM-ALK network. Network configurations that predispose NPM-ALK to optimally control proliferation (A) and cell survival (B) are depicted where the size of a node represents its effect on the endpoint—the smaller is the node, the smaller is the effect. The colour represents signal intensity—the darker the node, the more active it is when the control node is highly active.

SHP1 has an inhibitory effect on proliferation and cell survival. Its effect in the simulations is however masked by the downstream control node (NPM-ALK), whose activity in the simulations is increased without influence from SHP1’s state. To further validate our results, we repeated the same analysis using SHP1 as control node. We identified the active nodes in the network following an independent activity increase of SHP1, and we obtained a comparable outcome as with NMP-ALK (compare Fig 3 with S1 Fig). This suggests that perturbations of central network components such as NPM-ALK or SHP1 result in the activation of the same signalling pathways.

By comparing the p85/PI3K/p110 → PIP3 → pAKT and the GRB/SHC/IRS1 → VAV1 → CDC42 cascades, it is evident that they have different colour intensities (see Fig 3). In contrast, in S1 Fig, they have the same colour intensity. The size of the nodes for each endpoint is similar when comparing the two cascades with respect to proliferation (compare Fig 3A with S1A Fig) or to cell survival (compare Fig 3B with S1B Fig), which implies that they are activated in the same way when the control node is turned active. The difference in colour intensity observed between the two cascades in Fig 3 informs on the signal capacities of the cascades. In Fig 3, upon NPM-ALK activation, the nodes in GRB/SHC/IRS1 → VAV1 → CDC42 (darker) will reach higher activity than the ones in p85/PI3K/p110 → PIP3 → pAKT. In contrast, upon SHP1 activation, the nodes in both cascades exhibit similar dynamical ranges (similar node colour, see S1 Fig).

Node areas suggested that cell survival and proliferation are affected through NPM-ALK → p85/PI3K/p110 → PIP3 → pAKT (though to a somewhat smaller degree than NPM-ALK → JAK3/STAT3). It is indeed true that high activity of p85/PI3K/p110, PIP3 and pAKT will lead to cell survival and proliferation, but the flow analysis (node colour) indicates that this axis is not so often activated—alternative routes via CD30 (tumour necrosis factor receptor superfamily, member 8 (TNFRSF8)) seem to be more prevalent because the component nodes are more often highly active [56]. It also shows that changes in SHP1 activity cause a more homogeneous flow than changes in NPM-ALK, and that JAK3/STAT3 has a smaller influence on the endpoint when SHP1 activity increases.

In addition, form Fig 3 and S1 Fig it is evident that the GRB/SHC/IRS1 → VAV1 → CDC42 pathway is mainly involved in an increased activity of proliferation, while the Ras/MEK/ERK pathway affects principally cell survival (as previously shown experimentally [57]). This indicates that NPM-ALK can influence the endpoints through different routes of the signalling network. Importantly, the effect of NPM-ALK on the same endpoint is distributed over the signalling network and is not limited to one route only. This implies that therapeutic treatments targeting a single specific node of the network can fail if the network topology allows distributed signalling, and sheds light on the phenomenon of cancer resistance to drugs due to alternative signalling.

### Fine-Grained Simulations: Identification of Network Control Regions

The results of the coarse-grained simulations indicated several nodes of interest from a therapeutic point of view. Two of the nodes were studied in more detail, SHP1 and JAK3/STAT3. SHP1 was selected for its intrinsic inhibitory effects on cell survival and proliferation. The JAK3/STAT3 pathway was selected due to its significance based on results of the coarse-grained simulations, its reported relevance to ALK-mediated cancers [34, 41] and the availability of JAK3 and STAT3 inhibitors [49, 51, 52] which make it a valid therapeutic target. By systematically varying the activity of JAK3/STAT3 and SHP1 combined with different activity levels of NPM-ALK, we could identify regions in the parameter space of the system where small changes induce large effects on the endpoints. Such areas indicate a chaotic behaviour of the system.

**Fig 4:**
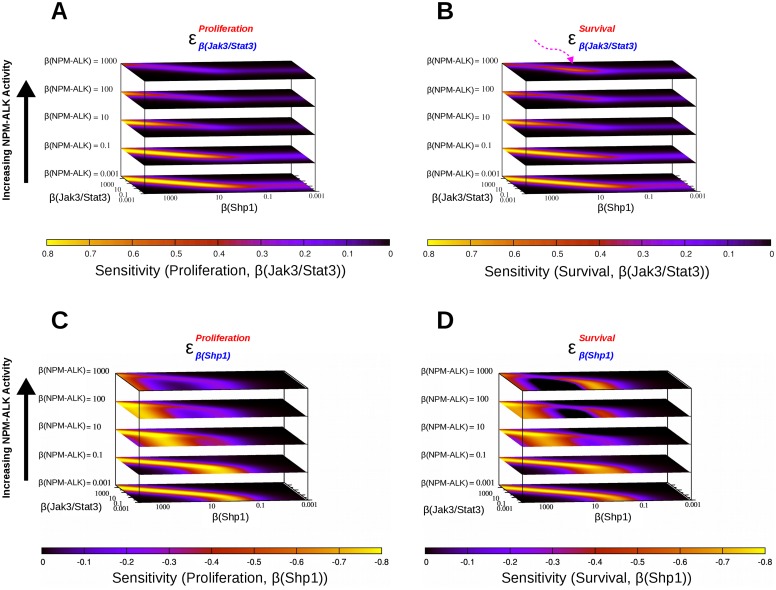
Sensitivity heat maps of the NPM-ALK network. Sensitivity maps were calculated in response to variations in JAK3/STAT3, SHP1 and NPM-ALK activities. Independent activity levels of JAK3/STAT3 and SHP1 are represented in the xy-plane, those of NPM-ALK are represented along the z-axis. Sensitivity of proliferation and cell survival calculated with respect to JAK3/STAT3 variations results in positive values (A and B, respectively). Values with respect to SHP1 variations, which only connects other network nodes by inhibiting links, are negative (C and D, respectively). The pink arrow in B highlights a region of increased sensitivity at high NPM-ALK activity. The sensitive areas in C and D (yellow) indicate a variable pattern of sensitive regions of the parameter space as a function of NPM-ALK activity. At intermediate NPM-ALK activity, these regions are larger than at low and high NPM-ALK activity. In addition, a bi-modal sensitivity pattern appears at high levels of NPM-ALK activity.

The effects of variations in JAK3/STAT3 activity on proliferation (*i.e.*, the sensitivity of proliferation with respect to changes in JAK3/STAT3 independent activity) decrease with increasing NPM-ALK (yellow areas in Fig 4A). The larger the activity of NPM-ALK, the larger are the values of both JAK3/STAT3 and SHP1’s activities where high sensitivity is observed. If one considers activities of NPM-ALK in the range set by *β* = 0.1 to 10, it appears that proliferation is not very sensitive to variations in JAK3/STAT3 activity unless both SHP1 and JAK3/STAT3 are (very) highly active. In general, low activity of SHP1 indicates that proliferation does not depend on JAK3/STAT3, unless NPM-ALK levels are low as well.

The effects of variations in JAK3/STAT3 activity on cell survival (Fig 4B) are very similar to those observed on proliferation. One notable exception is that a small region of increased sensitivity of cell survival appears, indicating that the system can regain controllability at high NPM-ALK with respect to changes in JAK3/STAT3 activity (see pink arrow in Fig 4B).

The sensitivity profiles of cell survival and proliferation with respect to variation in the activity of SHP1 are rather complex. When the activity of NPM-ALK is low (set by *β* = 0.001) or somewhat controlled (set by *β* = 0.1) the situation resembles the results for the sensitivity profiles with respect to JAK3/STAT3 (see the lower two surfaces in Fig 4C and 4D), except that the sensitivity to SHP1 is negative (yellow regions). At higher activities of NPM-ALK (set by *β* = 10 to 100) the pattern changes completely, and regions of high controllability (yellow region in Fig 4C and 4D) are visible where SHP1’s activity is high. These regions span almost completely the activity range of JAK3/STAT3. At the maximal activity range of NPM-ALK (highest surface of Fig 4C and 4D) the sensitivity pattern is more complex, and areas of negative sensitivity are observed also for the independent activity range 0.1 < *β* < 10 of SHP1. Altogether, analysis of the simulations reveals a complex relation between NPM-ALK, JAK3/STAT3, SHP1 and the endpoints. Regions of controllability (sensitivity which is far from zero) depend on NPM-ALK’s activity in a non-intuitive way.

## Conclusions

NPM-ALK-mediated signalling was studied through the use of a computational approach, taking into account and synthesizing via network analysis the current (partial) state of knowledge. The pathogenic end-states were particularly sensitive to the activities of the interleukins group considered in this study according to our current knowledge (*i.e.*, IL-2, IL-4, IL-7, IL-9, IL-15, IL-21 and IL-22) as well as to HSP-90, JAK3/STAT3 (activators) and SHP1 (inhibitor). Analysis of signal flow from NPM-ALK through the network revealed several alternative pathways through which the system may bypass inhibition (for example as a response to the exposure to drugs targeting the main signalling pathways used under physiologically benign conditions). These alternative pathways make it difficult to control signal transduction if NMP-ALK inhibition fails, *e.g.*, due to resistance mutations. Further analysis of JAK3/STAT3 and SHP1 was performed, and revealed that under some conditions it can be possible to keep the system under control through interventions in reducing JAK3/STAT3 signalling and increasing SHP1’s activity. Interestingly, some controllable areas in the parameter space were observed even at high levels of NPM-ALK activity. The simulations suggest that inhibition of the JAK3/STAT3 pathway and/or boosting the natural activity of SHP1 may be useful as alternatives to ALK inhibition. The simulations also reveal that a state of increased cell survival mainly employs the Ras/MEK/ERK pathway while the VAV1-CDC42 pathway is used to enhance cellular proliferation, suggesting targets for combined therapies. More quantitative experiments are needed to identify the conditions under which such treatments could have an effect.

## Methods

### Network Representation

A network model of NPM-ALK has been prepared combining the sparse experimental information found in the literature (*i.e.*, [29, 32, 37, 58, 59] and references therein). This information is analysed altogether as a single system and the factors that exert nonlinear control on the endpoints are studied with our network analysis program [38, 39]. Several oncogenic proteins interact with NPM-ALK. JAK/STAT and the cytokine system are found to be involved in multitude of cancers. Deregulated STAT3 is associated with ALK-positive ALCL [60–62] and is apparently needed for maintenance of the neoplastic phenotype [63]. NPM-ALK interacts with STAT3 by phosphorylating either STAT3 or its associated partner JAK3, leading to increased survival and proliferation [32, 64]. SHP1 has an inhibitory effect on NPM-ALK and JAK/STAT signalling that has been shown to potently attenuate the effect of cytokines [65, 66]. The p85/PI3K/p110 → PIP3 → pAKT pathway, as well as several lipid phosphatases that convert PIP3 back to PIP2, are involved in several processes that contribute to tumour development such as cell survival, proliferation, growth and motility [30, 37]. NPM-ALK and other ALK fusion proteins have also been shown to activate the Ras/MEK/ERK → AP-1 (also named JunB) cascade that lead to expression of CD30 involved in cell survival [29, 67–69]. In addition, interaction between NPM-ALK and the PLC-*γ* → IP3/DAG/Ca^2+^/PKC pathway, that connects to the Ras/MEK/ERK cascade, was also reported to be involved in mitogenic activity and proliferative response of Ba/F3 cells [31]. Interaction between HSP90 and NPM-ALK was reported where HSP90 was suggested to protect NPM-ALK from proteasome degradation [37, 70, 71]. Finally, the Ras/MEK/ERK cascade is also activated by the GRB/SHC/IRS1 effectors that lead to the activation of Ras [72–75] and VAV1-CDC42 path [57, 57, 76–79].

### Network Simulation and Sensitivity Analysis

Simulations have been performed according to the framework developed by us before [38, 39]. Briefly, the NPM-ALK network was built and analysed with a quantitative phenomenological modelling framework which relied on empirical Hill-type transfer functions as interaction links between nodes. This enabled the integration of experimental information in a flexible model based on simple yet sound formalism derived from classical enzyme kinetics. Signalling interactions were represented as a set of ordinary differential equations (ODEs) which was further simulated and analysed. The simulation procedure yielded steady-state activity levels of the different components in the network according to a given set of parameters. It consequently involved calculation of sensitivities corresponding to each parameter change. Both calculation of the steady-state activity and sensitivity analysis were carried out by use of parallel computational architectures thereby enabling the screening of a large number of conditions. This resulted in the identification of key control points of the studied network and enabled the testing of hypotheses on therapeutic strategies.

This computational approach allows efficient integration of sparse experimental information on signalling into network models. It requires only enough knowledge to set up qualitative boolean models (activation/inhibition networks) but provides quantitative insights on the studied system that take into account nonlinear regulatory effects such as feedbacks, pleiotropy and redundancy. The range of independent activities (*β*) that determine the individual nodes’ activities used in this study are summarised in S1 Table. See [38] for a complete list of parameters available in our code.

#### Sensitivity Analysis in Coarse-Grained Simulations

The coarse-grained simulations consist of exhaustively simulating all combinations of the NPM-ALK network states with high (*β* = 0.1) or low (*β* = 0.001) basal, independent activity (see S1 Table). Without considering the combined activity change of multiple control nodes simultaneously, but rather changes occurring subsequently one after another (as it would be expected by point mutations affecting the activity of a protein), this generates *s*^*n*^ = 2^20^ = 1 048 576 conditions (*s* is the number of states a node can assume, *n* is the number of nodes in the network) that are subsequently analysed for sensitivity: for each pair of simulated conditions that differ by a single parameter (*i.e.*, pair of simulations where the network states are identical except for a single node that is low in the first simulation and high in the second, or *vice versa*), the sensitivity of a node *N*_*i*_ with respect to another node *N*_*j*_’s independent activity (*β*(*N*_*j*_)) is calculated according to:

$$S\left(N_{i},\beta \left(N_{j}\right)\right)=\varepsilon_{\beta (N_{j})=low\;\to \;\beta \left(N_{j}\right)=high}^{SS{\left(N_{i}\right)}_{\beta (N_{j})=low}\;\to \;SS{\left(N_{i}\right)}_{\beta (N_{j})=high}}=\frac{ln\{\frac{SS{\left(N_{i}\right)}_{\beta (N_{j})=high}}{SS{\left(N_{i}\right)}_{\beta (N_{j})=low}}\}}{ln\{\frac{\beta (N_{j})=high}{\beta (N_{j})=low}\}}$$ (1)

where *SS*(*N*) denotes the steady-state activity of a node *N*. The arrow (→) indicates a change in condition.

This results in a set of calculated sensitivities derived from the coarse-grained simulations that comprises $s^{n}\cdot \frac{n}{s}\cdot \left(s-1\right)=2^{20}\cdot \frac{20}{2}\cdot 1\;=\;10\;485\;760$ sensitivity values. Note that this calculation follows the combinatorial distribution between the nodes limited by the number of states assumed by each node (here, two: high and low). Each of these values expresses the strength of a direct or indirect link between any two nodes in the network. For example if a node *X* is connected (directly or through intermediates) to a node *Y* by a positive sensitivity value (*S*(*X*, *β*(*Y*)) > 0), it means that upon an increase of *Y*’s activity, *X* will also increase (the higher the sensitivity the stronger the amplification of the signal form *Y* to *X*). Conversely, if *X*’s sensitivity with respect to *Y* is negative (*S*(*X*, *β*(*Y*)) < 0), *X* will decrease its activity upon an increase in *Y*’s activity. Finally, if *X* and *Y* are connected by a near-zero sensitivity value (*S*(*X*, *β*(*Y*)) ≈ 0), then the intermediate nodes between *X* and *Y* buffer the signal which results in *X* and *Y* being independent of each other.

#### Sensitivity Analysis in Fine-Grained Simulations

Fine-grained simulations consist of a more detailed study of the system explored by coarse-grained simulations. In these simulations, the majority of the network components are associated with low activity values (see S1 Table). Few nodes are modelled by simulating the network after initiating their activities within a given range, in small steps. This allows a more in-depth, quantitative understanding of the control nodes to the network endpoints (Fig 4). Simulations of steady-state activities as well as sensitivity analysis were performed according to the methods described in articles [38, 39].

#### Robustness

Activity changes in network nodes as parameters are varied (parametric uncertainty), as well as the variability of the network behaviour with respect to the number of nodes considered (structural uncertainty), were studied in our previous work. We showed that we could obtain consistent results both by setting single-valued parameters and by setting numerical ranges for each parameter screened, the width of the range reflecting the extent of variability. This showed that the method was robust against a wide range of parameter variation and reliable towards parametric uncertainty [38]. We also showed that equivalent results were obtained by the addition of ∼50% of nodes and links to a network (note that robustness tests consider a network able to tolerate variation of 5–20% in the number of the nodes as highly robust [80, 81]). This shows that the method is also robust with respect to structural uncertainty [39].

### Effect of Network States on the Endpoints

We denote as “*control nodes*” those that, upon their activity change (representing external or internal perturbations), would cause changes in the activity of the other nodes in the network. Cell proliferation and survival are denoted as the network’s “*endpoints*” and their activity is proportional to the pathological potential of a given network state.

Are there any preferred routes for a signal that drives cell survival or proliferation in our network model? To examine such potential routes, the proportions of the occurrence of *high* and *low* activity for each node in coarse-grained simulations were calculated when the endpoints were highly active. If a node has no correlation with an endpoint, the corresponding proportion is expected to be ∼50%. The larger the deviation from this proportion, the larger the involvement of the node within the network.

The **area of a node** in Fig 3 is proportional to its involvement in the network upon a change in the control node’s activity (the control node, which is NPM-ALK (Fig 3) or SHP1 (S1 Fig) undergoes 100-fold activity increase and is coloured in black). The closer is the ratio between the probabilities to find a node in high or low activity to 50%, the smaller is the node (represented using Cytoscape [82] by the node width ranging from a value of 30 to 120 corresponding to minimum to maximum of the calculated effect in the data set).

### Signal Intensity

Changes in the activity of any individual node (from low to high) influence not only the activity of the endpoints but also that of all other nodes. The average activity of any node *i* as a consequence of an activity change of the control node, *j*, is:

$$\hat{{ϒ}_{i,\beta (j)=high}}=\bar{SS_{i,\beta (j)=0.1}},j\neq i$$ (2)

where the bar denotes an average and *SS*_*i*,*β*(*j*)_ the steady-state of node *i* when the control node *j* is set to an independent activity of *β*(*j*). Similarly, $\hat{{ϒ}_{i,\beta (j)=low}}$ is calculated as:

$$\hat{{ϒ}_{i,\beta (j)=low}}=\bar{SS_{i,\beta (j)=0.001}},j\neq i$$ (3)

The ratio $\hat{{ϒ}_{i,\beta (j)=high}}/\hat{{ϒ}_{i,\beta (j)=low}}$ represents the effect of the control node’s independent activity change (*β*(*j*) = *low* → *high*) on the steady-state activity of any other node (*SS*_*i*_). This measure is represented in Fig 3 and S1 Fig as the **colour intensity of a node**: the darker the node, the higher is its $\hat{{ϒ}_{i,\beta (j)=high}}/\hat{{ϒ}_{i,\beta (j)=low}}$ ratio, *i.e.*, the more intense is the signal transduced through that node as a consequence of the control node’s activity change. The colour code in the network configuration diagrams ranges from 50 to 255 in RGB code corresponding to maximum to minimum of the calculated value in the data set, respectively, except for the control node that assumes a value of 0 in the RGB code (black in Fig 3 and S1 Fig). Note that this measure is scaled relative to the control node’s activity and is consequently relative to a single network diagram. Therefore, the colour code compares the nodes’ activity range similarly to the relative gene expression dynamic range of a gene in response to the overexpression of an upstream interacting gene.

## Supporting Information

S1 Table Values of the activity parameter (*β*) as used in the coarse-grained and fine-grained simulation setup. Activity units are arbitrary and relative. Only the initial set-up of NPM-ALK, JAK3/STAT3 and SHP1 is varied in the fine-grained simulations.
(PDF)

S1 Fig Signal flow in the NPM-ALK network (based on changes in SHP1 activity). Network configurations that predispose SHP1 to optimally control proliferation (A) and cell survival (B) are depicted similarly to Fig 3.
(TIFF)
