# Supplementary material for: Sensitivity Analysis of the NPM-ALK Signalling Network Reveals Important Pathways for Anaplastic Large Cell Lymphoma Combination Therapy
Source: PLoS One. 2016 Sep 26;11(9):e0163011. doi: 10.1371/journal.pone.0163011 (PMC5036789; doi:10.1371/journal.pone.0163011)
Supplement: S1 Table — Activity units are arbitrary and relative. Only the initial set-up of NPM-ALK, JAK3/STAT3 and SHP1 is varied in the fine-grained simulations. (PDF) [file pone.0163011.s001.pdf]

S1 Table

Parameter Name	Coarse-grained Simulations*	Fine-grained Simulations*
$\beta(AP1)$	10^{-3} - $10^{-1}(100)$	10^{-3}
$\beta(CD30)$	10^{-3} - $10^{-1}(100)$	10^{-3}
$\beta(CDC42)$	10^{-3} - $10^{-1}(100)$	10^{-3}
$\beta(GRB2/SHC/IRS1)$	10^{-3} - $10^{-1}(100)$	10^{-3}
$\beta(HSP90)$	10^{-3} - $10^{-1}(100)$	10^{-3}
$\beta(Interleukins)$	10^{-3} - $10^{-1}(100)$	10^{-3}
$\beta(IP3/DAG/Ca2/PKC)$	10^{-3} - $10^{-1}(100)$	10^{-3}
$\beta(JAK3/STAT3)$	10^{-3} - $10^{-1}(100)$	10^{-3} - $10^{+4}(1.2)$
$\beta(MEK/ERK)$	10^{-3} - $10^{-1}(100)$	10^{-3}
$\beta(NPM-ALK)$	10^{-3} - $10^{-1}(100)$	10^{-3} - $10^{+3}(10)$
$\beta(p85/PI3K/p110)$	10^{-3} - $10^{-1}(100)$	10^{-3}
$\beta(pAKT)$	10^{-3} - $10^{-1}(100)$	10^{-3}
$\beta(phosphatases(tens.\,hom.))$	10^{-3} - $10^{-1}(100)$	10^{-3}
$\beta(PIP3)$	10^{-3} - $10^{-1}(100)$	10^{-3}
$\beta(PLC\gamma)$	10^{-3} - $10^{-1}(100)$	10^{-3}
$\beta(Proliferation)$	10^{-3} - $10^{-1}(100)$	10^{-3}
$\beta(Ras)$	10^{-3} - $10^{-1}(100)$	10^{-3}
$\beta(SHP1)$	10^{-3} - $10^{-1}(100)$	10^{-3} - $10^{+4}(1.2)$
$\beta(Survival)$	10^{-3} - $10^{-1}(100)$	10^{-3}
$\beta(VAV1)$	10^{-3} - $10^{-1}(100)$	10^{-3}

* Range of Variation (Fold-Variation Step)
