# Supplementary figures and images for: Sensitivity Analysis of the NPM-ALK Signalling Network Reveals Important Pathways for Anaplastic Large Cell Lymphoma Combination Therapy
Source: PLoS One. 2016 Sep 26;11(9):e0163011. doi: 10.1371/journal.pone.0163011 (PMC5036789; doi:10.1371/journal.pone.0163011)

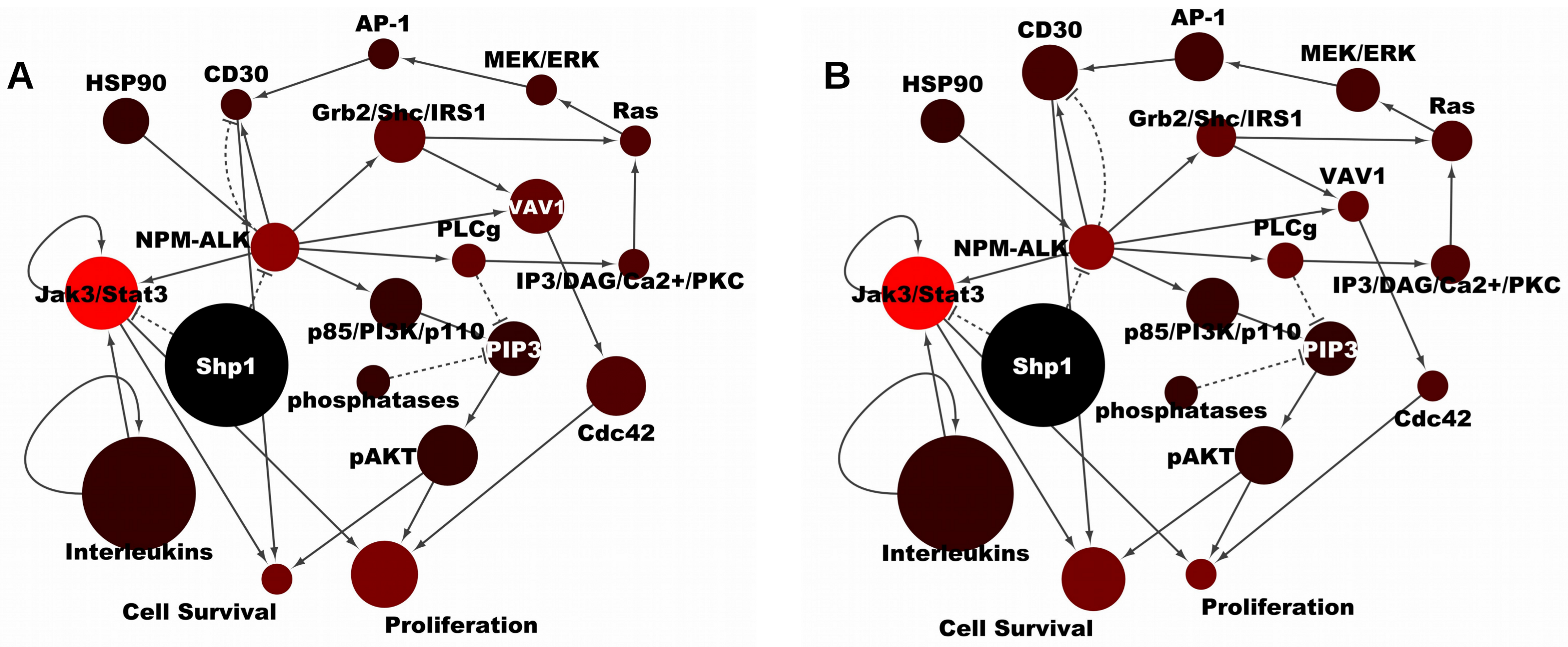

Supplement: S1 Fig — Network configurations that predispose SHP1 to optimally control proliferation (A) and cell survival (B) are depicted similarly to Fig 3. (TIFF) [file pone.0163011.s002.tiff]
